# Six-month binocular stereopsis recovery and its influencing factors in children with intermittent exotropia

**DOI:** 10.1186/s12886-024-03412-x

**Published:** 2024-03-27

**Authors:** Guohong Zhao, Jing Fu, Yue Qi, Yidi Wang, Wenbin Wei

**Affiliations:** Beijing Tongren Eye Center, Beijing key Laboratory of Intraocular Tumor Diagnosis and Treatment, Beijing Tongren Hospital, Capital Medical University, 100730 Beijing, China

**Keywords:** Intermittent exotropia, Stereopsis, Influencing factors, Children

## Abstract

**Objective:**

To investigate the recovery of binocular stereopsis recovery and its influencing factors in children with intermittent exotropia after successful correction of eye position.

**Methods:**

Prospective clinical study. A total of 178 patients, aged 9 ∼ 14 (10.8 ± 1.7) years, who were successfully corrected after intermittent exotropia surgery at the Beijing Tongren Hospital Affiliated to Capital Medical University from October 2023 to September 2023 were collected, the follow-up duration was six-month or longer. Paired t test, Pearson correlation analysis and multivariable linear regression analysis were used to probe preoperative clinical features that may predict the stereopsis six months after surgery.

**Results:**

Six months after surgery, the angle of deviation of the patients met the orthotopic standard, and there was significant difference compared with that before surgery (distant: -2.7^△^±3.2^△^ vs. -30.5^△^±8.4^△^, t=-25.3, *P* < 0.001. Near:-3.7^△^±4.1^△^ vs. -33.7^△^±8.0^△^, t=-26.1, *P* < 0.001). Distant stereopsis (3.0 ± 0.6 vs. 3.9 ± 0.4, t = 4.9, *P* < 0.05) and near stereopsis (2.3 ± 0.5 vs. 2.6 ± 0.4, t = 3.8, *P* < 0.05) were both significantly improved compared with that of before surgery. 17% and 22% patients rebuilt normal distant stereopsis and normal near stereopsis, respectively. Preoperative distant stereopsis (*r*=-0.26, *P* = 0.004) and near stereopsis (*r*=-0.23, *P* = 0.011) was significantly negatively correlated with convergence reserve. Multivariable analysis showed that patients’ age (β = 0.003, *p* = 0.037), anisometropia (β = 0.015, *p* = 0.043), and preoperative distant stereopsis (β = 0.456, *p* < 0.001) were significantly associated with postoperative distant stereopsis. Patients’ age (β = 0.005, *p* = 0.044), anisometropia (β = 0.127, *p* = 0.034), angle of deviation (β=-0.230, *p* = 0.020), and preoperative near stereopsis (β = 0.136, *p* < 0.001) were significantly associated with postoperative near stereopsis.

**Conclusion:**

IXT patients could get eye position fixed after surgery, about 20% patients benefited from stereopsis improvement. Patient’s age, binocular anisometropia, angle of deviation and preoperative stereopsis were independent factors influencing postoperative stereopsis.

## Introduction

Intermittent exotropia (IXT) is the most common type of clinical strabismus, the prevalence of which ranges from 0.1 to 3.7% in children and adolescents as reported by epidemiological studies [1–4]. IXT patients tend to maintain good binocular vision function. In addition, with eye position corrected by proper interventions, binocular vision functions such as perceptual fusion and stereovision may also be improved remarkably [5, 6].

However, the prognosis (long-term success rate) varies greatly among studies. Yang M [7] and coworkers reported that, 7.8 months after IXT surgery, only 35.6% of patients achieved normal stereoscopic vision. Lee MH H [8] and coworkers reported that 12 months after surgery, the success rate ranged from 46 to 70% depending on whether bilateral lateral rectus recession was performed. Lim SH [9] and coworkers reported a success rate of 58% at the one-year follow-up after surgery.

To improve surgical efficacy and benefit more patients, it is highly important to pay attention to the influencing factors of long-term prognosis, which are still unclear. This study analysed the binocular function status of 300 patients in the orthotopic eye position six months after IXT surgery after successful correction of strabismus and explored the correlation of this status with preoperative clinical characteristics; it provides a basis for future clinicians to predict postoperative binocular function status based on patients’ preoperative clinical characteristics and the need for early intervention in clinical decision-making.

## Methods

### Study design

This is a prospective consecutively cases series. 300 children and adolescents who underwent IXT surgery at the Beijing Tongren Hospital affiliated to Capital Medical University from October 2022 to September 2023. This study adheres to the Declaration of Helsinki and has been approved by the Medical Ethics Committee of the Beijing Tongren Hospital. All included patients and their guardians have signed informed consent forms.

### Inclusion and exclusion criteria

Inclusion criteria: (1) The distance and close range exophoria ≤ 10 ^△^ as measured using a prism and alternating cover test, or the internal phoria < 5 ^△^; (2) Best corrected visual acuity (BCVA) for both eyes ≥ 0.8 as measured by standard logarithmic visual acuity chart; (3) The anisometropia between two eyes is less than 1.50 diopter (D). 4)Follow-up period of 6 months or more.

Exclusion criteria: (1) Limited eye movement in a certain direction; (2) Combined vertical deviation ≥ 5 ^△^; (3) Those who still have diplopia when looking straight ahead; (4) The difference in BCVA between two eyes is more than 2 lines.

### Examinations

All patients were required to undergo routine eye examination, quantitative examination of strabismus, sensory fusion, and stereopsis before and after surgery. Besides, after surgery, the following examinations were also performed to better evaluate the binocular visual function, including fusion range, near point of convergence, accommodation amplitude, and accommodation flexibility. All tests require the patient to wear corrective lenses. The specific methods are as follows.

### Procedure of examinations

Routine eye examination: The subjective spherical equivalent error (SER) and BCVA were measured, the pupil was not dilated. The anterior segment was examined by slit-lamp microscopy and the fundus was examined by indirect fundus microscopy.

Quantitative examination of strabismus: The angle of deviation was measured using the prism and alternate cover test (PACT) at 33 cm for near and 6 m for distance fixations [10].

Worth 4 dot: Worth 4 dot was used to test whether patients have distant or near sensory fusion. Seeing four dots at the same time represents binocular perceptual fusion; Seeing two or three dots at the same time represents monocular suppression; Seeing five dots were considered diplopic.

Stereopsis: both distant stereopsis and near stereopsis were measured. Near stereopsis at near fixation was measured using random dot by Yan Shaoming. Yan Shaoming refers to a set of stereoscopic visual examination maps. The examination using Yan Shaoming is as followed. The patients were asked tor wear corrective lenses, with red and green lenses. The vertical distance between the examination map and the visual axis was 40 cm. Binocular vision at distance fixation, including simultaneous vision, fusion, and distance stereopsis, was examined by synoptophore (CLEMENT-CLARKE, UK, 2001).

The fusional vergence amplitude was measured using a horizontal prism bar, and an accommodative target was used first at distance fixation (3 m) and then at near fixation (1/3m) [11] with base-in (BI) for negative vergence and base-out (BO) for positive vergence. Measurement of the near point of convergence: patients were asked to look at an accommodative target located 40 cm away, and the examiner gradually moved the target toward the patient’s eyes until the patient reported that the target had become two targets. The distance between the break point and parallel point of the patient’s lateral canthus was measured.。.

The amplitude of accommodation was measured using the negative lens method. BCVA was measured first and the line was recorded as line A, then the patients were asked to focus on a single visual icon in the upper line of line A, then the spherical lenses were adjusted gradually by -0.25D.

Accommodative flexibility: patients were asked to read the “E” chart at 40 cm in sequence with a ± 2.00 D reverse lens within 1 min.

All the tests were performed after appropriate refractive correction. Each of these tests was performed before surgery and postoperatively.

### Statistical analysis

Stereoacuity was log transformed. Shapiro-test was used to check whether continuous variables were normally distributed, mean values and standardized deviations were used for basic statistical description of continuous variables. Paired t-test was used for comparison on continuous variables before and after surgery. Chi-square test was used for comparison on categorical variables. Pearson correlation test was used to evaluate the relationship between preoperative clinical features and postoperative stereoacuity, minus value means negative correlation between variables. Multivariable linear regression analysis was used to evaluate the influencing factors of postoperative stereoacuity.The significance level was set to be 0.05, two tailed. The open source R program (https://www.r-project.org/, version 4.3.2) was used for data analysis.

## Results

### Characteristics of IXT patients

A total of 178 patients with IXT were included, including 97 males and 81 females. Age: 9 ∼ 14 (10.8 ± 1.7) years old. The average SER of the right eyes and left eyes were − 1.375 ± 1.25 D, and − 1.250 ± 1.31 D respectively. The median duration of disease course of IXT was 4.5 years (range: 0.9 to 10.3 years). The median follow-up duration was 10 months (Range: 6 to 13 months). The strabismus angle of deviation measured after surgery all met the criteria of normotopia, and were significantly different from that measured before surgery (distant: -2.7^Δ^±3.2^Δ^ vs. -30.5^Δ^±8.4^Δ^, t=-25.3, *P* < 0.001. Near:-3.7^Δ^±4.1^Δ^ vs. -33.7^Δ^±8.0^Δ^, t=-26.1, *P* < 0.001). Distant stereopsis (3.0 ± 0.6 vs. 3.9 ± 0.4, t = 4.9, *P* < 0.05) and near stereopsis (2.3 ± 0.5 vs. 2.6 ± 0.4, t = 3.8, *P* < 0.05) were both significantly improved compared with that of before surgery(Table 1). 17% and 22% patients rebuilt normal distant stereopsis and normal near stereopsis, respectively.

**Table 1:** Comparison of angle of deviation and binocular stereopsis pre- and 6-months postoperative

Time	strabismus angle, PD		Stereopsis (log arcsec)	
	Distance	Near	Distant	Near
Preoperation	-30.5 ± 8.4	-33.7 ± 8.0	3.9 ± 0.4	2.6 ± 0.4
6 months postoperation	-2.7 ± 3.2	-3.7 ± 4.1	3.0 ± 0.6	2.3 ± 0.5
*t/Z*	-25.3	-26.1	4.9	3.8
*P*	< 0.001	< 0.001	< 0.05	< 0.05

There was no correlation between preoperative clinical features and convergence reserve (*P* > 0.05), while preoperative distant stereopsis (*r*=-0.26, *P* = 0.004) and near stereopsis (*r*=-0.23, *P* = 0.011) was significantly negatively correlated with convergence reserve. This shows that the better the stereopsis before surgery, the stronger the near convergence reserve after surgery (Table 2). No more multiple analysis would be done since only one independent variable was correlated with convergence reserve.

**Table 2:** Correlation between preoperative clinical characteristics and 6-months postoperative distant convergence reserve and near convergence reserve in intermittent exotropia patients

Preoperative parameters	Distant convergence reserve		Near convergence reserve	
	*r*	*P*	*r*	*P*
Age, year	0.07	0.597	0.06	0.155
Gender	0.12	0.209	0.11	0.321
Myopia	-0.08	0.522	0.02	0.796
Disease duration, year	-0.02	0.895	-0.06	0.211
Anisometropia, D	0.03	0.809	0.04	0.565
Angle of deviation, PD				
Distance	0.08	0.556	0.07	0.301
Near	-0.03	0.722	-0.05	0.166
∆ Angle of deviation, PD	-0.11	0.221	-0.07	0.633
Stereopsis (log arcsec)				
Distant	0.02	0.588	-0.26	0.004
Near	-0.03	0.345	-0.23	0.011
Amplitude of accommodation	0.08	0.533	0.04	0.677

### Correlation between preoperative clinical characteristics and convergence reserve 6 months after surgery in intermittent exotropia patients

Distant stereopsis showed a significant association with near convergence reserve (*r*=-0.24, *p* = 0.007) (Table 2). Near stereopsis showed a significant association with near convergence reserve (*r*=-0.21, *p* = 0.003). No other preoperative clinical characteristics showed correlation with convergence reserve.

### Correlation analysis between preoperative clinical parameters and stereopsis 6 months after surgery in intermittent exotropia patients

As shown in Table 3, preoperative clinical parameters, including age, and anisometropia were positively correlated with distant stereopsis 6 months after surgery (*P* < 0.05). Myopia was positively correlated with both distant stereopsis and near stereopsis (*p* < 0.05). While age, degree of anisometropia, distant angle of deviation, were all significantly associated with near stereopsis 6 months after surgery (*P* < 0.05). Preoperative distant stereopsis and near stereopsis were positively correlated with stereopsis 6 months after surgery (*P* < 0.05).

**Table 3:** Correlation between preoperative clinical characteristics and 6 months postoperative distant stereopsis and near stereopsis in intermittent exotropia patients

Preoperative parameters	Postoperative Distant Stereopsis		Postoperative Near Stereopsis	
	r	*P*	r	*P*
Age, year	0.19	0.038	0.24	0.004
Gender	-0.01	0.152	0.07	0.323
Myopia	0.17	0.025	0.237	0.014
Disease duration, year	0.09	0.057	-0.03	0.354
Anisometropia, D	0.27	0.011	0.39	< 0.001
Angle of deviation, PD				
Distant	0.04	0.277	-0.21	0.021
Near	0.02	0.522	-0.17	0.212
∆ Angle of deviation, PD	0.04	0.655	0.04	0.331
Stereopsis (log arcsec)				
Distant	0.35	< 0.001	0.44	< 0.001
Near	0.31	< 0.001	0.48	< 0.001
Amplitude of accommodation	0.04	0.422	0.03	0.211

### Multivariable analysis of influential factors of postoperative stereopsis

Multivariable analysis (Table 4) was further performed since there are many factors correlated with postoperative stereopsis (Table 3). Results showed that patients’ age (β = 0.003, *p* = 0.037), anisometropia (β = 0.015, *p* = 0.043), and preoperative distant stereopsis (β = 0.456, *p* < 0.001) were significantly associated with postoperative distant stereopsis.

**Table 4:** Multivariable analysis of influential factors of postoperative stereopsis

Preoperative parameters	Postoperative Distant Stereopsis		Postoperative Near Stereopsis	
	β	*P*	β	*P*
Intercept	1.521	-	1.203	-
Age, year	0.003	0.037	0.005	0.044
Myopia	0.007	0.411	-0.005	0.539
Disease duration, year	0.007	0.066	-	-
Anisometropia, D	0.015	0.043	0.127	0.034
Angle of deviation (Distant)	-	-	-0.230	0.020
Stereopsis (log arcsec)				
Distant	0.456	< 0.001	-0.004	0.585
Near	-0.004	0.577	0.136	< 0.001

Formula to predict postoperative distant stereopsis (Y):

$$\begin{array}{l}{\rm{Y}}\,{\rm{ = }}\,{\rm{1}}{\rm{.521}}\,{\rm{ + }}\,{\rm{0}}{\rm{.003}} * {\rm{Age}}\,{\rm{ + }}\,{\rm{0}}{\rm{.015}} * {\rm{Anisometropia}}\\{\rm{ + }}\,{\rm{0}}{\rm{.456}} * {\rm{preoperative}}\,{\rm{distant}}\,{\rm{stereopsis}}\end{array}$$

Patients’ age (β = 0.005, *p* = 0.044), anisometropia (β = 0.127, *p* = 0.034), angle of deviation (β=-0.230, *p* = 0.020), and preoperative near stereopsis (β = 0.136, *p* < 0.001) were significantly associated with postoperative near stereopsis.

Formula to predict postoperative near stereopsis (Y):

$$\begin{array}{l}{\rm{Y}}\,{\rm{ = }}\,{\rm{1}}{\rm{.203}}\,{\rm{ + }}\,{\rm{0}}{\rm{.005}} * {\rm{Age}}\,{\rm{ + }}\,{\rm{0}}{\rm{.127}} * {\rm{Anisometropia}}\,\\{\rm{ - }}\,{\rm{0}}{\rm{.230}} * {\rm{Angle}}\,{\rm{of}}\,{\rm{deviation}}\,{\rm{ + }}\,{\rm{0}}{\rm{.136}} * {\rm{preoperative}}\,{\rm{near}}\,{\rm{stereopsis}}\end{array}$$

## Discussion

Stereopsis embodies the perception ability of binocular three-dimensional space vision, which is an advanced visual function based on binocular simultaneous vision and perceptual image fusion. This study explored the stereopsis of children aged 9–14 years after surgery through a median 6-month follow-up and explored the factors influencing the effect of stereopsis. The main findings of this study were as follows: (1) The overall stereoscopic function of the patients in this study improved after surgery; however, only 17% of the patients achieved normal distant stereopsis, and 22% of the patients achieved normal near stereopsis. (2) Patient age, binocular anisometropia, angle of deviation and preoperative stereopsis were found to be independent factors influencing postoperative stereopsis.

Preoperative binocular vision function is often used to assess the severity of the disease [12–14], and postoperative binocular vision function reflects the level of visual function repair [15, 16]. Theoretically, as IXT patients have a certain basis for binocular vision function, when the eye position of IXT patients is corrected after surgery, binocular vision function is significantly improved [17–19]. The results of this study are consistent with this viewpoint and those of previous studies. However, it is worth mentioning that the proportion of patients with normal stereoscopic function was not high, and nearly half of the patients in the present study could not recognize the maximum distance parallax pattern. This finding was different from the results of Yang et al. [7] and Pinele et al. [19], where more than 35% of patients were reported to exhibit normal stereoacuity.

The present study further analysed the correlation between preoperative clinical features and postoperative distant and near stereopsis and further verified this correlation by using multivariable models. Patient age, anisometropia, and preoperative stereopsis were correlated with postoperative outcomes. In other words, the older the patient, the greater the degree of anisometropia between the eyes and the worse the postoperative recovery from stereovision. The better the preoperative distance and near stereopsis, the better the postoperative stereopsis recovery. The development of binocular vision function in individuals with stereopsis is related to age. After surgical correction of the eye position during the critical period of development, the patient’s visual function can be further remodelled and repaired. Therefore, it is believed that the younger the patient, the greater the chance of binocular stereopsis recovery after surgery [20, 21]. In the present study, the average age of the participants was 10.2 years, which means that a large percentage of the children had good stereoscopic vision, which partially explains why only approximately 20% of the children benefited from stereopsis improvement. The timing of treatment is critical. For patients with the normal eye position, abnormal binocular parallax caused by anisometropia is the main cause of stereovision [22, 23]. Therefore, for the treatment of IXT, it is not enough to consider only the eye position; anisometropia is also important. Our study showed that anisometropia indeed matters in predicting long-term prognosis after adjustment for confounding factors in multivariate analysis. Moreover, preoperative stereoscopic function reflects the ability of the visual centre to control the right position of the eye [24]; thus, the preoperative status of stereoscopic function is another crucial factor for predicting long-term prognosis. Our results support this hypothesis. Yildirim C [25] and coworkers concluded that patients with better preoperative stereoscopic vision would have a greater success rate of postoperative eye position correction and more obvious improvement in stereopsis.

The fusion convergence reserve reflects the ability of a patient to reach fusional vergence and is also a reflection of the strength of the patient’s control of their eye position. It is also considered as an indicator for assessing the severity of the disease before surgery [12]. However, studies on the fusion convergence reserve in patients after surgery are rare. Wakayama A [26] and coworkers reported that the postoperative fusion convergence reserve of IXT patients was positively correlated with their ability to maintain phoria at both distance and near distance, indicating that the greater the fusion convergence reserve was, the greater the ability to maintain phoria after surgery. In this study, postoperative near-fusion convergence reserve was significantly correlated with preoperative distant and near stereoscopic vision, indicating that patients had good stereoscopic function before surgery and could obtain better fusion convergence reserve after surgery.

In conclusion, IXT patients can maintain their eye position after surgery, and binocular vision functions such as stereopsis can be significantly improved via this approach. However, a large percentage of patients suffer from abnormal stereovision functions after surgery. Patient age, anisometropia between the eyes, angle of deviation, and preoperative stereoscopic function largely determine postoperative stereoscopic function.

## Data Availability

The datasets used and/or analysed during the current study available from the corresponding author on reasonable request.
